# Effects of Isomaltulose Ingestion on Thermoregulatory Responses during Exercise in a Hot Environment

**DOI:** 10.3390/ijerph18115760

**Published:** 2021-05-27

**Authors:** Junto Otsuka, Yumi Okamoto, Naoto Fujii, Yasuaki Enoki, Daisuke Maejima, Takeshi Nishiyasu, Tatsuro Amano

**Affiliations:** 1 Laboratory for Exercise and Environmental Physiology, Faculty of Education, Niigata University, 8050, Igarashi-Ninocho, Nishiku, Niigata 950-2181, Japan; z20j013e@mail.cc.niigata-u.ac.jp (J.O.); z20j011j@mail.cc.niigata-u.ac.jp (Y.O.); 2 Faculty of Health and Sport Sciences, University of Tsukuba, Tsukuba City 305-8574, Japan; fujii.naoto.gb@u.tsukuba.ac.jp (N.F.); nishiyasu.takeshi.fw@u.tsukuba.ac.jp (T.N.); 3 Advanced Research Institutes, Bourbon Corporation, 316-2, Higashijima, Akihaku, Niigata 956-0841, Japan; enoki-yas@bourbon.co.jp (Y.E.); maejima-dai@bourbon.co.jp (D.M.)

**Keywords:** palatinose, high-intensity exercise, thermoregulation, rehydration, dehydration, sweat, exercise performance, sports nutrition, hydration, glycemic index

## Abstract

Isomaltulose is a low glycemic and insulinemic carbohydrate available as a constituent of sports drinks. However, it remains unclear whether thermoregulatory responses (sweating and cutaneous vasodilation) after isomaltulose drink ingestion differ from those of sucrose and water during exercise in a hot environment. Ten young healthy males consumed 10% sucrose, 10% isomaltulose, or water drinks. Thirty-five minutes after ingestion, they cycled for fifteen minutes at 75% peak oxygen uptake in a hot environment (30 °C, 40% relative humidity). Sucrose ingestion induced greater blood glucose concentration and insulin secretion at the pre-exercise state, compared with isomaltulose and/or water trials, with no differences during exercise in blood glucose. Change in plasma volume did not differ between the three trials throughout the experiment, but both sucrose and isomaltulose ingestions similarly increased plasma osmolality, as compared with water (main beverage effect, *p* = 0.040)—a key response that potentially delays the onset of heat loss responses. However, core temperature thresholds and slopes for heat loss responses were not different between the trials during exercise. These results suggest that ingestion of isomaltulose beverages induces low glycemic and insulinemic states before exercise but does not alter thermoregulatory responses during exercise in a hot environment, compared with sucrose or water.

## 1. Introduction

Consumption of fluids containing carbohydrates and electrolytes is recommended before and during exercise in a hot environment to maintain muscle glucose metabolism and fluid balance as well as physiological function, including thermoregulatory and cardiovascular responses [1,2,3]. Conventional sports drinks contain carbohydrates such as glucose, fructose, maltodextrin, and sucrose. Isomaltulose (ISO) is a naturally occurring disaccharide composed of α-1, 6-linked glucose, and fructose, in contrast to the α-1, 2-glycosidic bond found in sucrose (SUC). ISO is fully digestible, with a relatively low glycemic index (32) as compared to sucrose (65). Both are available in sports drinks [4,5]. Several studies have investigated physiological responses following ISO ingestion during exercise, including glycemic and insulinemic responses, substrate utilization, and exercise performance [6,7,8,9,10]. However, despite the importance for sports drink consumption in a hot environment, ISO’s influence on thermoregulatory heat loss responses, including sweating and cutaneous vasodilation during heat stress in exercise, is unknown.

Studies have shown that blood glucose and insulin concentrations are lower following ISO ingestion, compared with SUC, until approximately 60 min after ingestion [5,11,12,13]. Slow glycemic and insulinemic responses to ISO may disturb heat loss responses by altering the state of plasma volume (PV) and plasma osmolality (P_osm_), both of which are important factors in modulating heat loss responses. It has been shown that PV expansion augments thermoregulatory sensitivity for heat loss responses (defined as the slopes for sweating and cutaneous vasodilation against core temperature elevation) [14,15,16,17], whereas P_osm_ elevation increases the core temperature threshold for these responses [18,19,20].

Water is passively absorbed with glucose and fructose in the small intestine. Thus, delayed glycemic response from ISO ingestion may also delay water absorption and, thereby, PV expansion. Delayed insulin secretion may also delay sodium and water reabsorptions in kidney tubules and, thereby, potentially attenuate PV expansion [21,22]. In addition, it is known that carbohydrate drink ingestion increases P_osm_, compared with water ingestion [23]. However, insulin secretion following carbohydrate ingestion is thought to lower the core temperature threshold for heat loss responses, possibly offsetting the elevated core temperature threshold associated with high P_osm_ [23]. Thus, it is believed that carbohydrate ingestion delays the core temperature threshold for heat loss responses only with drinks that produce low insulinemic responses [23].

SUC ingestion would establish higher glycemic and insulinemic responses, as compared with ISO and water ingestions ~30 min after fluid ingestion, and the response would sustain for an additional ~20 min at a resting state [5,11,12,13]. Therefore, to evaluate the possible negative effects of ISO ingestion (e.g., disturbance of heat loss responses), if any, the exercise duration should be within the period of desired glycemic and insulinemic states. This is thought to be corresponding to 30–50 min after fluid ingestion—the period in which low glycemic and insulinemic responses are typically seen with ISO, compared with SUC ingestion [5,11,12,13]. A high-intensity exercise protocol (e.g., more than 70% VO_2peak_) would allow optimal analysis of core temperature threshold and slope for heat loss responses [24] even in a short period of exercise by inducing sufficient thermoregulatory responses.

The aim of this study was to investigate the effect on thermoregulatory responses of ingesting carbohydrate–electrolyte beverages containing ISO before exercise in a hot environment, compared with that of ingesting beverages containing SUC and water. We hypothesized that ISO ingestion would disturb heat loss responses during exercise at 75% VO_2peak_ for 15 min in a hot environment, represented by a delayed core temperature threshold and an attenuated slope for sweating and cutaneous vasodilation, as compared with water and/or SUC. It has been reported that ISO ingestion may [6] or may not [7,8,9] have ergogenic effects on performance during prolonged exercise, compared with other carbohydrates; this effect has not been tested for short periods of exercise in a hot environment. It is known that the performance of final minute of exercise during 15–30 min competition typically seen in 5000 m and 10,000 m athletic events is important in determining the winner of the race [25]. Therefore, to provide additional insights regarding the influence of pre-exercise ISO ingestion, we investigated exercise performance following ISO ingestion during the final minute of the 15 min exercise as a secondary purpose of the study. As ergogenic effects of carbohydrate supplementation should be evident during exercise lasting at least 1 h [26], we hypothesized that ISO and SUC ingestion would not enhance power output in a performance trial, compared with water, during the final minute of a 15 min high-intensity exercise in a hot environment. It is important to note that the analysis of core temperature threshold and slope for heat loss responses requires initial thermoregulatory responses during exercise and thus the short period of sub-maximum exercise (14 min) and 1 min performance test never compromise the primary outcomes.

## 2. Materials and Methods

### 2.1. Ethical Approval

This study was approved by the Human Ethical Committee of Niigata University and was conducted in accordance with the latest version of the Declaration of Helsinki. Verbal and written informed consent were obtained from all participants prior to commencement of the experimental sessions.

### 2.2. Participants

Ten healthy and physically trained young men participated in the study (age: 20.4 ± 0.5 years, height: 1.74 ± 0.06 m, body mass: 67.1 ± 7.7 kg, peak oxygen uptake (VO_2peak_): 66.5 ± 6.9 mL kg^−1^ min^−1^). The participants had engaged in regular physical training for at least 2 h per day and 5–6 days per week for at least the past 4 years. They were recruited from athletic club in Niigata University. None of the participants were taking prescription medications and all were non-smokers, all of which were confirmed verbally when we obtained the informed consent.

We determined a minimum sample size of *n* = 10 would be required to detect an attenuation of core body temperature threshold for heat loss responses following carbohydrate drink ingestion with at least 80% statistical power (α = 0.05). This was based on an effect size of 1.00 calculated from an estimated reduction in core temperature threshold for heat loss responses after carbohydrate drink ingestion [23] (~0.2 °C) with SD of 0.2.

In a screening session, VO_2peak_ was determined during a ramp cycling protocol (18 W/min, 60 rpm) using a cycle ergometer (Powermax V3; Konami, Tokyo, Japan) in a temperate environment (25 °C and 50% relative humidity) until voluntary exhaustion. Oxygen consumption was determined using a breath-by-breath gas exchange measurement system (AE300S; Minato Medical Science, Osaka, Japan). All trials were conducted between November 2019 and May 2020 at Niigata University, Niigata, Japan. We did not include female participants, given known sex-related differences in sweating and cutaneous vasodilation [27,28] and female-specific modulation of hydration status related to the menstrual cycle [29].

### 2.3. Test Beverages

During the experimental session (see below), participants consumed 500 mL of one of three test beverages: flavored (Sunett^®^; Mitsubishi corporation life sciences, Tokyo, Japan) water (CON) and beverages containing carbohydrates–electrolytes consisting of 10% SUC and 10% ISO, respectively. Carbohydrate concentration was determined based on a previous study that reported an efficacy of isomaltulose ingestion on exercise performance and glycemic response [6]. The nutrients composition of each beverage is outlined in Table 1. Beverages were provided in clear plastic bottles, maintained at 37 °C to minimize any influences on thermoregulatory responses [30]. All beverages were matched color and flavor as well as sweetness by adding artificial sweetener (Sunett^®^) to CON (0.025%, *w*/*w*). It is known that a consumption of Sunett^®^ (acesulfame K) does not induce glycemic and insulin responses [31]. Participants were instructed to complete ingestion within 10 min.

### 2.4. Experimental Protocol

Three experimental trials were separated by a minimum of 6 days in a randomized, single-blind, cross-over design. Participants were instructed to refrain from consuming alcohol or caffeine and from participating in strenuous physical activity for at least 24 h prior to each trial. Additionally, the night before each trial, standard meal was provided as a lunchbox (Makunouchi-bento L size; Plenus, Tokyo, Japan), consisting of ~132 g of carbohydrate, ~32 g of fats, and ~34 g of protein, with an energy equivalent of ~921 kcal. This included 500 mL of non-caffeine Japanese tea. In addition, participants were asked to consume 500 mL of water before going to bed. At least 2 h before the start of the trial, participants were instructed to consume a light breakfast, consisting of a cup of jelly and two energy bars (Calorie mate: Otsuka pharmaceutical, Tokyo, Japan), consisting of ~33 g of carbohydrate, 11.2 g of fat, 4.3 g of protein, and 0.5 g of salt; energy equivalent of 200 kcal) as well as 500 mL water.

**Figure 1:**
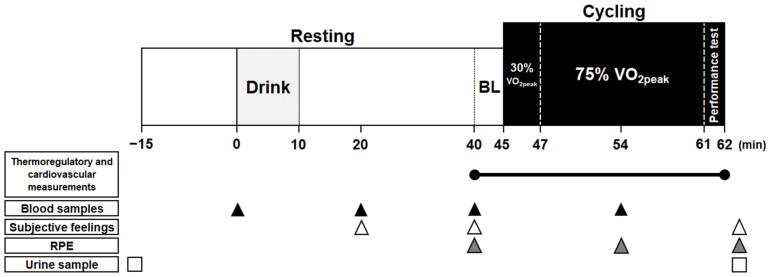
Schematic timeline of experimental protocol. RPE, rate of perceived exertion; BL, baseline.

A schematic experimental protocol is shown in Figure 1. Participants reported to the laboratory between 9:00 a.m. and 10:00 a.m. Upon arrival, urine samples were collected to assess hydration status, as determined by urine specific gravity [32]. Height was assessed using a stadiometer (YS501-P; Sanyu, Tokyo, Japan). Participants dressed in running shorts and entered the environmental chamber, maintained at 30 °C and 40% relative humidity. Body mass, wearing the shorts only, was then measured. After resting in a semi-recumbent position for approximately 15 min, the first blood sample was collected. Participants then rested for an additional 45 min, and during the first 10 min, they consumed one of three randomly assigned test beverages. The second and third blood collections were performed at 20 and 40 min, respectively, after fluid consumption commenced. Instrumentations were conducted during the 40 min rest, after commencement of fluid consumption. Baseline (BL) measurements for thermoregulatory and cardiovascular variables were obtained during the final 5 min of the 45 min resting period. After BL measurements, participants sat on a cycle ergometer and commenced the exercise trial, which consisted of a warming-up period at 30% VO_2peak_ for 2 min and then sub-maximum exercise at 75% VO_2peak_ for 14 min. This was followed by assessing power output during 1 min of maximal-effort cycling at a pedal resistance of 7 kP/kg of body mass. Participants were verbally encouraged to achieve their maximum effort during this period. Cadence was set at 60 rpm during warming-up and sub-maximum exercise. Blood samples were collected after 7 min of exercise at 75% VO_2peak_. After the cycling trial was completed, sweat was carefully wiped off before assessing body mass, wearing the running shorts only, and urine samples were collected.

### 2.5. Measurements

Blood samples were collected from the fingertip, in a semi-recumbent position at rest, before and after fluid consumption, and in a cycling position during exercise. We did not employ larger blood sampling (e.g., from antecubital vein) because we were not medically qualified to do the procedure. We collected ~450 μL of blood sample at rest and smaller volumes (~150 μL) during exercise. This was due to the limited sampling period (~2 min) during exercise, as it was difficult to maintain high-intensity cycling for more than a few minutes without holding the handlebar. Measurements of hemoglobin concentration (Hb), hematocrit (Hct), plasma osmolality (P_osm_), plasma sodium concentration (P(Na^+^)), plasma potassium concentration (P(K^+^)), plasma chlorine concentration (P(Cl^−^)), and insulin concentration (P_ins_), as well as blood glucose (Glu) and lactate (Lac) concentrations, were obtained from each sample. The sampling procedure was fixed between individuals such that we assessed variables in the order of Hb and Glu followed by Lac. Immediately thereafter followed blood collection into two capillaries (EM mystar hematocrit capillary; As one, Osaka, Japan) for Hct, followed by blood collection into a microtube (MBS capillary; Micro blood science, Tokyo, Japan) for P_ins_ and electrolytes. The blood plasma in the capillaries was used for osmolarity analysis. All assessments were performed in duplicate. Owing to the limited blood collected, we were not able to assess P(Na^+^), P(K^+^), P(Cl^−^), and P_ins_ during exercise. Hb was measured using a spectrophotometric device (Hemocue Hb 201; HemoCue, Angelholm, Sweden). Hct was determined using a microhematocrit method. PV (ΔPV), BV, and cell volume (CV) were determined using the procedures specified by Dill and Costill [33]. For measurement of P_osm_, P(Na^+^), P(K^+^), P(Cl^−^), and P_ins_, blood samples were centrifuged, and the extracted plasma was frozen at −80 °C until analysis. P_osm_ was measured using the freezing point depression method (Fiske 210 Micro Osmometer; Advanced instruments, MA), and P_ins_ was measured using a sandwich ELISA method (YK060; Yanaihara, Fujinomiya, Japan). The intra-assay coefficient of variation for P_ins_ was 4.0 ± 3.6% in the present study. Glu (Glu-Test Every, SKK, Nagoya, Japan), Lac (Lactate Pro 2 LT-1730; Arkray, Kyoto, Japan), P(Na^+^), P(K^+^), and P(Cl^−^) (STAX-5 Inspire, Techno medica, Yokohama, Japan) concentrations were determined using portable analyzers. P_ins_, Glu, Lac, P(Na^+^), P(K^+^), and P(Cl^−^) were corrected for changes in PV [34].

Rectal temperature (T_re_) was measured using a thermistor probe (401J; Nikkiso-Thermo, Tokyo, Japan), inserted 12 cm past the anal sphincter. Skin temperature was measured by a thermistor (ITP082-25; Nikkiso-Thermo, Tokyo, Japan) affixed to four skin sites on the chest, upper arm, thigh, and lower leg. Mean skin temperatures (T_sk_) were calculated as follows: [35] chest, 30%; upper arm, 30%; thigh, 20%; and lower leg, 20%. Rectal and local skin temperatures were recorded at 1 s intervals using a data storage device (Model N543; Nikkiso-Thermo, Tokyo, Japan).

Local sweat rates on the forearm and chest were measured using the ventilated capsule method according to the methods used in our laboratory and described elsewhere [36,37]. A 3.14 cm^2^ plastic capsule was affixed using topical glue (Collodion; Kanto chemical, Tokyo, Japan). Dry nitrogen gas was passed through each capsule over the skin surface at a rate of 1.3 L/min. Water content from the effluent air was measured using a capacitance hygrometer (HMP60; Vaisala, Helsinki, Finland). Skin blood flow (SkBF) on the chest was measured continuously by laser-Doppler velocimetry (FLO-C1; Omegawave, Tokyo, Japan); a laser-Doppler probe was placed adjacent to the ventilated capsule on the chest. Heart rate (HR) was recorded using a Polar coded WearLink and transmitter and the RS800 interface (Polar Electro Oy, Kempele, Finland).

Urine volume and urine specific gravity were assessed using a graduated cylinder and a refractometer (UG-D; Atago, Tokyo, Japan), respectively. Whole body sweat loss was assessed from changes in body mass before and after the trial, accounting for the weight of the beverage consumed. Power output was recorded at 10 Hz to evaluate exercise performance based on mean and peak power outputs during the final 1 min of maximum exercise. Participants were asked to report subjective feelings relating to thirst, stomach fullness, and hunger at 20 and 40 min after beverage ingestions as well as at post-exercise. They also evaluated the palatability of the beverage immediately after consumption. These questionnaires were a 10 cm visual analog scale, with 0 cm representing “not at all” and 10 cm representing “very” [38]. Ratings of perceived exertion (RPE) and comfort/thermal sensations were assessed using a 6–20 Borg scale [39] and a Gagge scale [40], respectively, at BL, during exercise (at the seventh minute of 75% VO_2peak_ intensity), and after exercise.

### 2.6. Data and Statistical Analyses

All variables recorded continuously were averaged for 5 min at BL and for each minute during the exercise trial. SkBF was calculated as a percentage of BL. T_re_ thresholds and slopes for the changes in sweat rate, and SkBF during exercise, were assessed using the segmented regression analysis method [24].

For all blood measurements, a two-way repeated-measures analysis of variance (ANOVA) was performed on the repeated factors of protocol stage (four stages: pre-fluid ingestion, 20 and 40 min after ingestion, and exercise; or three stages (without exercise)), and on the test beverage (three stages: CON, SUC, and ISO). For thermoregulatory variables, a two-way repeated-measures ANOVA was performed on the repeated factors of the protocol stage (BL and 1 min intervals during exercise) and on the test beverage. Thirst, stomach fullness, and hunger were evaluated using a two-way repeated-measures ANOVA with the repeated factors of the protocol stage (three stages: 20 and 40 min after fluid ingestion and post-exercise) and with the test beverage. Maximum and peak power outputs, palatability, body mass loss, urine volume, urine specific gravity, and T_re_ (ΔT_re_) thresholds for sweating and cutaneous vasodilation were evaluated between the conditions using one-way repeated-measures ANOVA. The Geisser–Greenhouse correction was applied if the assumption of sphericity had been violated. A normal distribution was confirmed on the basis of Q–Q plot assessment. Post hoc analysis was performed using Tukey’s multiple comparisons test. Three participants who showed excessive elevations in P_ins_ beyond the measurable range were excluded from the analysis of P_ins_. One participant was excluded from the data analysis for rectal and skin temperatures, as well as for P_osm_, due to measurement failures. Data are presented as mean ± SD, and statistical significance was set at 0.05.

## 3. Results

### 3.1. Hematologic Variables

**Figure 2:**
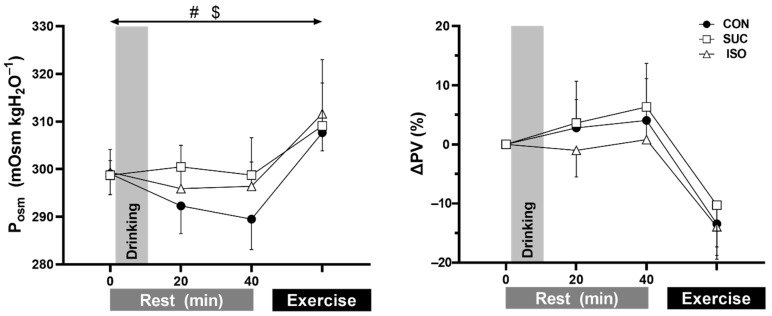
Plasma osmolality (P_osm_, *n* = 9) and change in plasma volume (ΔPV, *n* = 10) at rest and during exercise at the seventh minute of 75% peak oxygen uptake. CON, control (water); SUC, sucrose; ISO, isomaltulose. Values are presented as mean ± SD. #, *p* = 0.006 group difference in beverage between SUC vs. CON. $, *p* = 0.003 group difference in beverage between ISO vs. CON.

P_osm_ and ΔPV are presented in Figure 2. A significant main effect of beverage consumption was observed in P_osm_ (*p* = 0.040), which was higher in SUC and ISO than in CON (*p* = 0.006 and *p* = 0.003, respectively). No interaction effect for beverage and time was observed in P_osm_ (*p* = 0.105). No main effect of beverage type or interaction effect of beverage and time was observed in ΔPV (*p* = 0.101 and *p* = 0.309, respectively) or in absolute BV, CV, PV, or Hct (Table 2). A main effect of beverage type was observed in Hb (*p* = 0.042), such that it was higher in ISO than in SUC and CON (both *p* ≤ 0.001) (Table 2).

**Figure 3:**
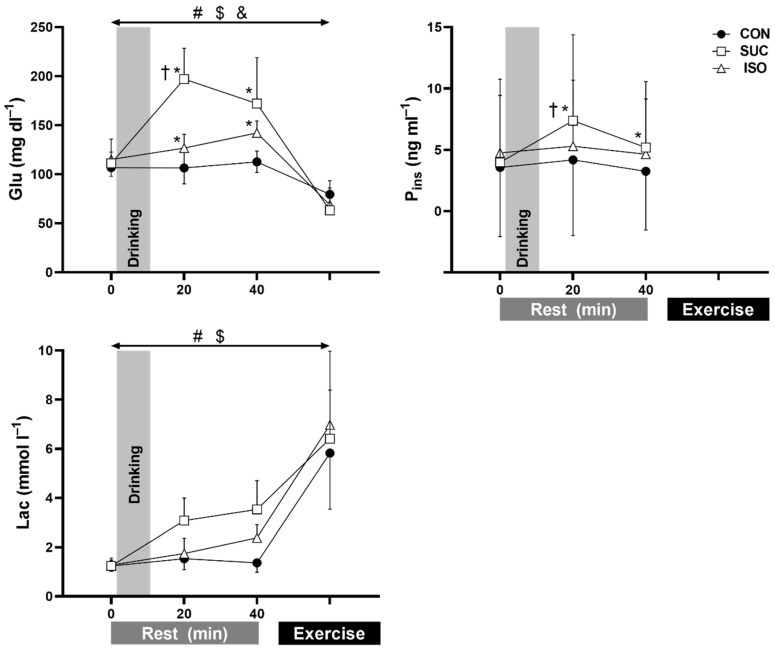
Blood glucose (Glu, *n* = 10), plasma insulin (P_ins_, *n* = 7), blood lactate (Lac, *n* = 10) concentrations at rest and during exercise at the seventh minute of peak oxygen uptake. Insulin data are missing because of limited blood sample volume. CON, control (water); SUC, sucrose; ISO, isomaltulose. Values are presented as mean ± SD. * *p* ≤ 0.031 vs. CON at each time point; †, *p* ≤ 0.047 vs. ISO at each time point. #, *p* ≤ 0.001 group difference in beverage between SUC vs. CON; $, *p* ≤ 0.035 group difference in beverage between ISO vs. CON. &, *p* = 0.006 group difference in beverage between SUC vs. ISO.

Glu, P_ins_, and Lac are presented in Figure 3. A main effect of beverage type and a significant interaction of beverage and time were observed in Glu (both *p* < 0.001). Pre-exercise Glu was higher in SUC and ISO than in CON at both 20 and 40 min (all *p* ≤ 0.023) and was higher with SUC than with ISO at 20 min (*p* ≤ 0.001) after fluid ingestion. However, Glu was not different during exercise (*p* ≥ 0.280). A significant interaction of beverage and time (*p* = 0.017) and a main effect of time (*p* = 0.022) but not beverage (*p* = 0.076) were observed in P_ins_. Pre-exercise P_ins_ was higher in SUC than in ISO (*p* = 0.047) and CON (*p* = 0.031) at 20 min (7.37 ± 6.99, 5.29 ± 5.38, and 4.18 ± 6.17 ng mL^−1^ for SUC, ISO, and CON, respectively) and in CON (*p* = 0.017) but not in ISO (*p* = 0.689) at 40 min after fluid ingestion (5.17 ± 5.37, 4.63 ± 4.51, and 3.33 ± 4.71 ng mL^−1^, respectively). A significant main effect of beverage type was observed in Lac (*p* = 0.008), with higher Lac in SUC and ISO than in CON (*p* < 0.001 and *p* = 0.035, respectively). No interaction effect of beverage and time was observed in Lac (*p* = 0.087). Neither a main effect of beverage type nor an interaction effect of beverage and time was observed in P(Na^+^), P(K^+^), and P(Cl^−^).

### 3.2. Thermoregulatory and Cardiovascular Variables

**Figure 4:**
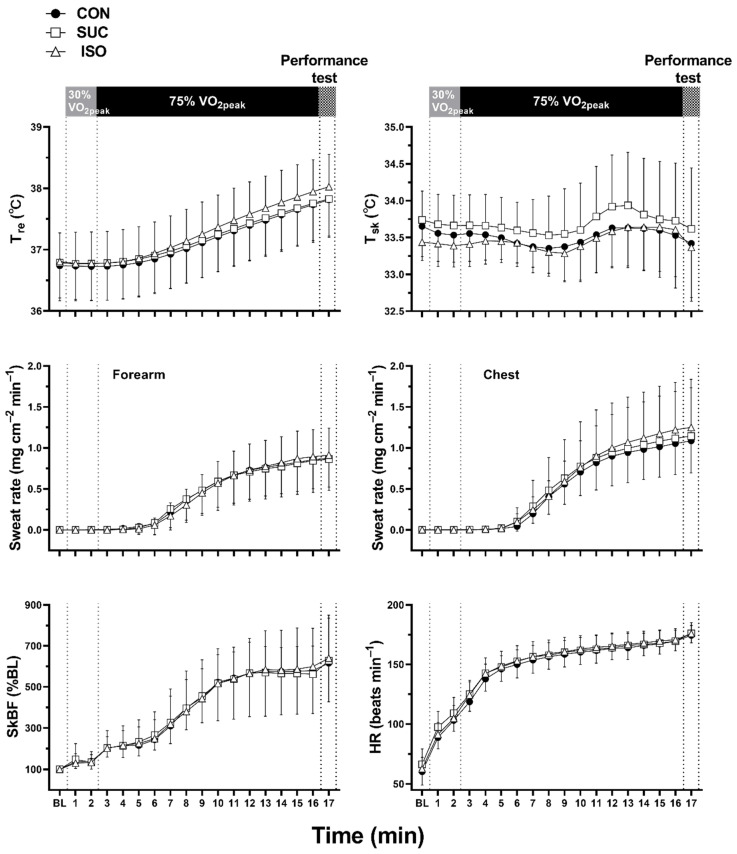
Rectal temperature (T_re_, *n* = 9), mean skin temperature (T_sk_, *n* = 9), sweat rate on the forearm and chest, skin blood flow (SkBF) on the chest, and heart rate (HR) during exercise. CON, control (water); SUC, sucrose; ISO, isomaltulose; BL, baseline. Values are presented as mean ± SD. *n* = 10, unless otherwise indicated.

**Figure 5:**
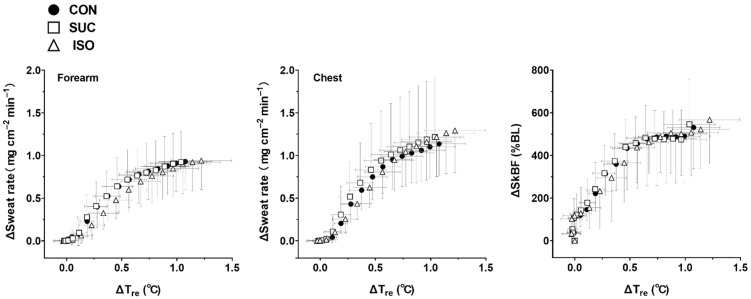
Changes in sweat rate on the forearm and chest, and skin blood flow (SkBF) on the chest, as a function of change in rectal temperature (ΔT_re_) during exercise. CON, control (water); SUC, sucrose; ISO, isomaltulose; BL, baseline. Values are shown as means ± SD. *n* = 9 for all variables.

Responses in T_re_, T_sk_, forearm and chest sweat rate, chest SkBF, and HR were not different between trials (effect of beverage type and interaction with time, all *p* ≥ 0.100; Figure 4). In addition, T_re_ thresholds (all *p* ≥ 0.269) and slopes (all *p* ≥ 0.418) for sweat rate on the chest and forearm, as well as SkBF on the chest, were not different between trials (Figure 5 and Table 3).

### 3.3. Body Mass Loss and Urinary Output

Absolute body mass loss (0.79 ± 0.12, 0.79 ± 0.24, and 0.68 ± 0.17 kg for CON, SUC, and ISO, respectively; *p* = 0.340) and relative body mass loss (1.19% ± 0.46%, 1.34% ± 0.32%, and 1.1% ± 0.21%; *p* = 0.283) were similar between the trials. Urine volume, measured after exercise, did not differ between trials (294 ± 195, 287 ± 116, and 174 ± 124 mL for CON, SUC, and ISO, respectively; *p* = 0.098). Similarly, urine specific gravity on arrival (1.011 ± 0.007, 1.013 ± 0.009, and 1.014 ± 0.011 for CON, SUC, and ISO, respectively; *p* = 0.686) and after exercise (1.009 ± 0.007, 1.010 ± 0.008, and 1.018 ± 0.008; *p* = 0.064) did not differ between trials.

### 3.4. Subjective Feelings

Palatability of the beverage was higher in SUC than in CON (*p* = 0.045), whilst it was not different in ISO as compared with other drinks (*p* = 0.073 and 0.821 for vs. CON and SUC, respectively). Other subjective variables were not different between trials (Table 4).

### 3.5. Exercise Performance

Mean power output (337 ± 76, 345 ± 71, and 332 ± 55 W for CON, SUC, and ISO, respectively; *p* = 0.518) and peak power output (during the final minute of performance) (380 ± 98, 407 ± 98, and 371 ± 63 W; *p* = 0.179) were not different between the trials.

## 4. Discussion

We observed greater glucose and insulin concentrations pre-exercise in SUC than in ISO and CON. Ingestion of ISO and SUC did not induce pre-exercise PV expansion, but it did elevate P_osm_, compared with that of CON—a key response known to delay core temperature threshold for sweating and cutaneous vasodilation [18,19,20,41]. Despite these hematologic responses following ISO and SUC ingestion, established at pre-exercise resting state in a hot environment, core temperature threshold and slope for sweating and cutaneous vasodilation during exercise were similar between trials. This suggests that ingesting beverages containing ISO with electrolytes prior to exercise does not disturb thermoregulatory responses during exercise in a hot environment. This is an important insight for the use of ISO in sports drinks during exercise in a hot environment.

Initially, we expected several factors to affect thermoregulatory responses during exercise, through modulation of PV and P_osm_ following ISO and SUC ingestion. Regarding PV, different glycemic response between SUC and ISO would influence the rate of water absorption and thereby PV expansion—a response known to augment thermoregulatory sensitivity for heat loss [14,15,16,17]. Insulin secretion may also aid PV expansion by promoting sodium and fluid reabsorption in kidney tubules [21,22]. However, PV expansion and thermoregulatory sensitivity for sweating and cutaneous vasodilation were similar among our trials, despite pre-exercise glucose concentration and insulin secretion being higher with SUC ingestion than with ISO or CON. As for P_osm_, although we observed elevated P_osm_ following both SUC and ISO ingestion, such differences did not affect core temperature threshold for sweating and cutaneous vasodilation during exercise. Because insulin secretion following SUC ingestion may attenuate core temperature threshold for heat loss responses [23], there is a possibility that, in the SUC trial, insulin secretion might counteract the delaying effect of hyperosmolality on the core temperature threshold for heat loss. However, as ISO ingestion did not affect the core temperature threshold for heat loss, compared with CON ingestion, despite elevation of P_osm_ without alteration of insulin secretion, P_osm_ elevation and insulin secretion in the SUC trial may not interact in modulating core temperature threshold for heat loss responses.

The precise reason for absence of modulating heat loss responses following ISO and SUC ingestion during exercise is unclear. We assume that hematologic responses at the pre-exercise state, following ISO and SUC ingestion, did not translate into thermoregulatory responses during exercise. Indeed, it has been suggested that an elevated plasma Glu concentration after carbohydrate ingestion rapidly decreases to hypoglycemic levels within 10–20 min [42]. This response may occur in both high and low glycemic foods, whilst the magnitude of the response is either similar between these foods [43] or greater in high glycemic food [44]. The insulin response at the initial stage of the exercise is thought to be similar between low and high glycemic foods despite the differences in pre-exercise state [43,44]. We therefore assume that the glucose concentration (Figure 3) and probably insulin response might not differ between ISO and SUC during exercise, thereby abolishing its effect on thermoregulatory responses in the present study. In addition, although P_osm_ was higher in SUC and ISO trials than in CON (a significant effect of the trial), this response was likely diminished during exercise (Figure 2). Thus, it seems that elevation of P_osm_, induced by a high-intensity exercise [45], might override the changes in P_osm_ following carbohydrate ingestion, which could potentially suppress heat loss responses [18,19,20,41]. It is thought that the altered hematological state associated with carbohydrate beverage ingestion at pre-exercise rest does not necessarily modulate thermoregulatory responses during exercise.

It has been suggested that ingesting isomaltulose could induce gastrointestinal discomfort, thereby attenuating exercise performance [8]; however, this response is not necessarily reported [7]. We develop these observations by showing that ISO ingestion does not influence subjective feelings as compared with CON and SUC at rest and during exercise even in the heat. Palatability was higher in SUC as compared with CON. However, assumingly, the difference in palatability might not affect the overall physiological responses and performance during exercise since exercise was conducted 35 min after the fluid ingestion.

As expected, we did not observe any ergogenic effects of ISO or SUC on exercise performance in a hot environment. This was probably because the exercise duration was too short for performance enhancement through carbohydrate ingestions, as has been described elsewhere [26]. It would be debatable to employ exercise performance assessment for 1 min only in a carbohydrate supplementation study. We employed this protocol since this is a similar time-dependent exercise to a real-world competition, such as a 5000 m athletic event [25], albeit it is not exactly the same. In addition, the primary aim of the present study was to assess potential negative effects of ISO on thermoregulation during a specific narrow time window following the fluid ingestion (see introduction). Thus, it was difficult to assess exercise performance for more than 1 min to complete the exercise within our target time window. We assumed that glycemic response (and thus related hydration status) would change during a prolonged exercise such that blood glucose concentration would start to increase in ISO as compared with SUC. Thus, there would be a possibility that the effects of ISO ingestion on exercise performance in the heat, if any, would be pronounced in other performance tests during a prolonged exercise. Associated with this, König et al. [6] reported an efficacy of ISO ingestion on exercise performance during exercise for approximately 30 min after 90 min sub-maximum trial. Further studies are required to determine whether ISO ingestion prior to or during prolonged exercise in the heat would improve performance.

### Limitations

First, we focused on thermoregulatory responses during 15 min of exercise following 35 min of rest after ISO ingestion. This was an obligatory exercise protocol as we had expected that ISO ingestion would have negative effects on thermoregulatory responses during the specific exercise period (see Introduction) [5,11,12,13]. Some people would argue that an exercise period of 15 min is too short for evaluating thermoregulatory responses. However, it is important to note that the 15 min exercise at 75% VO_2peak_ was of sufficient duration to analyze core temperature threshold and slope for heat loss responses (Figure 4 and Figure 5). This is because the current analysis offers the initial thermoregulatory responses during exercise only. Therefore, the short exercise duration combined with a 1 min performance test did not compromise the primary findings in the present study. Nevertheless, it remains unknown whether ISO ingestion would affect thermoregulatory responses during prolonged exercise lasting ~120 min, during which time blood glucose and insulin concentrations would be higher in ISO than in SUC [5,11,12,13]. Second, as we could not measure insulin concentrations during exercise, owing to small blood samples, we could not confirm the insulin response. In addition, as we did not perform the oral glucose tolerance test for screening the participants, we could not predict the abnormal insulin secretion in some participants. This resulted in a reduction of the sample size (*n* = 7) for insulin analysis. Furthermore, despite the acceptable coefficient of variation for P_ins_ (4%), we observed relatively large SD for insulin measurement. Hence, due to a reduced sample size and large SD in P_ins_, care should be taken in interpreting the outcomes of this variable in the present study.

Third, participants were not low glycemic status before test drink consumption (Figure 3), probably because they ate a light breakfast 2 h before the participation. It remains unknown if and how the glycemic status before participation affected the results in the present study. Fourth, we did not measure maximum SkBF and blood pressure, both of which are recommended to be assessed for better understanding of its neural control [46]. Hence, care should be taken in interpreting our SkBF data. Fifth, as we assessed VO_2peak_ in thermoneutral conditions in the preliminary test; there might be a possibility that the relative intensity during exercise in a hot environment was higher than 75% VO_2peak_ in the present study. Finally, we employed a single-blind experiment due to our limited laboratory staff who could fully engage in the experiments. Whilst we carefully performed the experiments and analysis to exclude any biases, the single-blind trial could potentially limit the interpretation of current findings.

## 5. Conclusions

We showed that pre-exercise ingestion of a beverage containing ISO did not disturb thermoregulatory responses during exercise in a hot environment, compared with that of a beverage containing SUC and CON.

## Figures and Tables

**Table 1:** Nutrient component in the test drinks.

Nutrient Component	CON	SUC	ISO
Energy (kcal 100 mL^−1^)	0	40	40
Carbohydrate (mmol L^−1^)	0.0	292.1	292.1
Sodium (mmol L^−1^)	20.6	20.6	20.6
Calcium (mmol L^−1^)	1.2	1.2	1.2
Magnesium (mmol L^−1^)	1.0	1.0	1.0
Potassium (mmol L^−1^)	4.5	4.5	4.5
Osmolality (mOsmol kg^−1^)	62	454	403

**Table 2:** Hematologic variables at rest and during exercise in each trial.

		Rest			Exercise	*p*		
		Pre-Ingestion	Time into Ingestion			Time	Beverage	Time × Beverage
			20 min	40 min				
BV (mL)	CON	100	101 ± 3	100 ± 5	90 ± 5	<0.001	0.076	0.192
	SUC	100	102 ± 5	105 ± 5	93 ± 5			
	ISO	100	99 ± 2	100 ± 3	90 ± 3			
CV (mL)	CON	46 ± 2	45 ± 2	44 ± 2	42 ± 3	0.001	0.350	0.060
	SUC	46 ± 2	46 ± 2	47 ± 4	44 ± 3			
	ISO	46 ± 2	46 ± 2	46 ± 2	44 ± 2			
PV (mL)	CON	53 ± 2	55 ± 3	55 ± 3	46 ± 3	<0.001	0.087	0.322
	SUC	54 ± 2	55 ± 4	57 ± 3	48 ± 4			
	ISO	53 ± 2	53 ± 3	54 ± 3	46 ± 3			
Hct (%)	CON	46 ± 2	45 ± 2	44 ± 2	47 ± 2	<0.001	0.309	0.410
	SUC	46 ± 2	45 ± 2	45 ± 2	48 ± 3			
	ISO	46 ± 2	46 ± 2	46 ± 2	49 ± 2			
Hb (g dL^−1^)	CON ^$^	13.9 ± 0.7	13.8 ± 0.7	13.8 ± 0.6	15.4 ± 0.9	<0.001	0.042	0.214
	SUC ^$^	14.3 ± 1.0	14.0 ± 1.1	13.6 ± 1.1	15.3 ± 1.2			
	ISO	14.4 ± 0.8	14.5 ± 0.8	14.4 ± 0.8	15.9 ± 0.9			
P(Na^+^) (mmol L^−1^)	CON	143.0 ± 2.3	144.3 ± 7.6	148.1 ± 11.2	-	0.069	0.085	0.179
	SUC	143.1 ± 2.2	146.3 ± 10.5	151.4 ± 12.1	-			
	ISO	143.2 ± 2.8	140.7 ± 6.7	141.7 ± 5.0	-			
P(K^+^) (mmol L^−1^)	CON	4.4 ± 0.5	4.7 ± 0.6	4.7 ± 0.8	-	0.296	0.272	0.176
	SUC	4.5 ± 0.5	4.3 ± 0.7	4.2 ± 0.4	-			
	ISO	4.5 ± 0.5	4.2 ± 0.4	4.8 ± 1.2	-			
P(Cl^−^) (mmol L^−1^)	CON	106.6 ± 3.0	106.9 ± 5.7	108.9 ± 8.3	-	0.168	0.225	0.147
	SUC	105.3 ± 2.3	108.7 ± 8.9	111.4 ± 8.0	-			
	ISO	107.0 ± 3.0	104.1 ± 5.6	105.7 ± 4.4	-			

Exercise measurements were performed at 7 min after the initiation of exercise at 75%VO_2peak_. BV, blood volume; CV, cell volume; PV, plasma volume; Hct, hematocrit; Hb, hemoglobin; P(Na^+^), plasma sodium; P(K^+^), plasma potassium; P(Cl^−^), plasma chloride; CON, control (water); SUC, sucrose; ISO, isomaltulose. Changes in volume (mL) for BV, CV, and PV were calculated based on pre-exercise baseline resting BV normalized at 100 mL [33]. Data for P(Na^+^), P(K^+^), and P(Cl^−^) are missing because of our limited blood sample volume. Values are presented as means ± SD. *n* = 10 for all measurements. ^$^, *p <* 0.001 group difference in beverage vs. ISO.

**Table 3:** Rectal temperature thresholds and slopes for sweating and cutaneous vasodilation in each trial.

			Threshold (°C)	*p*	Slope (mg cm^−2^ min^−1^/°C or %BL/°C)	*p*
T_re_	Δ Sweat rate on forearm	CON	36.81 ± 0.57	0.909	2.66 ± 2.43	0.453
		SUC	36.89 ± 0.64		2.33 ± 1.63	
		ISO	36.93 ± 0.53		1.62 ± 0.90	
	Δ Sweat rate on chest	CON	36.83 ± 0.56	0.904	3.11 ± 3.97	0.600
		SUC	36.81 ± 0.60		2.89 ± 2.33	
		ISO	36.92 ± 0.55		1.88 ± 0.66	
	Δ SkBF on chest	CON	36.83 ± 0.59	0.791	1597 ± 1351	0.516
		SUC	37.02 ± 0.58		1110 ± 276	
		ISO	37.98 ± 0.57		1127 ± 920	
ΔT_re_	Δ Sweat rate on forearm	CON	0.06 ± 0.07	0.269	2.61 ± 2.3	0.418
		SUC	0.11 ± 0.11		2.30 ± 1.62	
		ISO	0.13 ± 0.10		1.56 ± 0.86	
	Δ Sweat rate on chest	CON	0.07 ± 0.07	0.301	3.11 ± 3.97	0.606
		SUC	0.11 ± 0.08		2.89 ± 2.33	
		ISO	0.13 ± 1.10		1.88 ± 0.66	
	Δ SkBF on chest	CON	0.11 ± 0.06	0.276	1621 ± 1337	0.426
		SUC	0.16 ± 0.11		1201 ± 379	
		ISO	0.19 ± 0.13		1091 ± 834	

T_re_, rectal temperature; SkBF, skin blood flow; CON, control (water); SUC, sucrose; ISO, isomaltulose. Values are presented as means ± SD. *n* = 9 for all variables.

**Table 4:** Subjective feelings at rest and after exercise in each trial.

		Rest		Exercise	Post-Exercise	*p*		
		Time into Ingestion						
		20 min	40 min (BL before Exercise)			Time	Beverage	Time × Beverage
Hunger	CON	3.9 ± 2.0	4.3 ± 2.2	-	4.6 ± 2.5	0.449	0.416	0.440
	SUC	4.1 ± 2.7	4.2 ± 2.9	-	4.1 ± 3.1			
	ISO	3.5 ± 1.9	3.7 ± 2.2	-	3.9 ± 2.5			
Thirst	CON	1.4 ± 1.2	2.0 ± 1.8	-	4.6 ± 3.1	<0.001	0.394	0.541
	SUC	2.4 ± 2.0	2.8 ± 2.4	-	5.0 ± 3.4			
	ISO	2.8 ± 2.9	2.9 ± 2.8	-	5.2 ± 2.9			
Stomach fullness	CON	4.6 ± 2.2	3.8 ± 1.8	-	3.3 ± 1.9	<0.087	0.528	0.218
	SUC	4.0 ± 2.5	3.9 ± 2.5	-	3.8 ± 2.3			
	ISO	3.5 ± 2.4	3.3 ± 2.4	-	3.0 ± 2.4			
RPE	CON	-	7.5 ± 2.5	15.3 ± 2.2	18.8 ± 1.1	<0.001	0.407	0.463
	SUC	-	7.3 ± 1.9	14.3 ± 1.6	18.8 ± 1.0			
	ISO	-	7.9 ± 2.1	15.1 ± 2.2	18.9 ± 1.1			
Thermal sensation	CON	-	1.6 ± 0.7	3.2 ± 0.4	3.6 ± 0.5	<0.001	0.171	0.594
	SUC	-	1.4 ± 0.7	3.1 ± 0.7	3.5 ± 0.7			
	ISO	-	1.8 ± 0.8	3.1 ± 0.6	3.7 ± 0.5			
Comfort	CON	-	4.6 ± 1.1	6.6 ± 0.5	6.9 ± 0.3	<0.001	0.194	0.597
	SUC	-	4.3 ± 0.9	6.6 ± 0.5	6.8 ± 0.4			
	ISO	-	4.8 ± 1.0	6.7 ± 0.5	7.0 ± 0.0			
Palatability of the beverage	CON	4.4 ± 1.7	-	-	-	Main effect of beverage 0.020		
	SUC	6.7 ± 3.1 *	-	-	-			
	ISO	4.9 ± 3.0	-	-	-			

Exercise measurements were performed at 7 min after initiation of exercise at 75% peak oxygen uptake. Post-exercise assessment was performed immediately after exercise. CON, control (water); SUC, sucrose; ISO, isomaltulose; RPE, rate of perceived exertion. Values are presented as mean ± SD. *n* = 10 for all variables. * *p* = 0.045 vs. CON.

## Data Availability

The data that support the findings of this study are available from the corresponding author upon reasonable request.

## References

[1] Shirreffs S. (2009). Hydration in sport and exercise: Water, sports drinks and other drinks. Nutr. Bull..

[2] Coyle E.F., Montain S.J. (1992). Benefits of fluid replacement with carbohydrate during exercise. Med. Sci. Sports Exerc..

[3] Coyle E.F., Montain S.J. (1992). Carbohydrate and fluid ingestion during exercise: Are there trade-offs?. Med. Sci. Sports Exerc..

[4] Sentko A., Willibald-Ettle I., O’Donnell K., Kearsley M. (1988). Isomaltulose. Sweeteners and Sugar Alternatives in Food Technology.

[5] Sawale P.D., Shendurse A.M., Mohan M.S., Patil G. (2017). Isomaltulose (palatinose)–An emerging carbohydrate. Food Biosci..

[6] König D., Zdzieblik D., Holz A., Theis S., Gollhofer A. (2016). Substrate utilization and cycling performance following palatinose™ ingestion: A randomized, double-blind, controlled trial. Nutrients.

[7] Miyashita M., Hamada Y., Fujihira K., Namura S., Sakazaki M., Miyasaka K., Nagai Y. (2019). The effects of isomaltulose ingestion on gastric parameters and cycling performance in young men. J. Exerc. Sci. Fit..

[8] Oosthuyse T., Carstens M., Millen A.M. (2015). Ingesting isomaltulose versus fructose-maltodextrin during prolonged moderate-heavy exercise increases fat oxidation but impairs gastrointestinal comfort and cycling performance. Int. J. Sport Nutr. Exerc. Metab..

[9] Stevenson E.J., Watson A., Theis S., Holz A., Harper L.D., Russell M. (2017). A comparison of isomaltulose versus maltodextrin ingestion during soccer-specific exercise. Eur. J. Appl. Physiol..

[10] Achten J., Jentjens R.L., Brouns F., Jeukendrup A.E. (2007). Exogenous oxidation of isomaltulose is lower than that of sucrose during exercise in men. J. Nutr..

[11] Maeda A., Miyagawa J.I., Miuchi M., Nagai E., Konishi K., Matsuo T., Tokuda M., Kusunoki Y., Ochi H., Murai K. (2013). Effects of the naturally-occurring disaccharides, palatinose and sucrose, on incretin secretion in healthy non-obese subjects. J. Diabetes Investig..

[12] Holub I., Gostner A., Theis S., Nosek L., Kudlich T., Melcher R., Scheppach W. (2010). Novel findings on the metabolic effects of the low glycaemic carbohydrate isomaltulose (palatinose™). Br. J. Nutr..

[13] Kawai K., Okuda Y., Yamashita K. (1985). Changes in blood glucose and insulin after an oral palatinose administration in normal subjects. Endocrinol. Jpn..

[14] Fortney S.M., Nadel E.R., Wenger C.B., Bove J.R. (1981). Effect of blood volume on sweating rate and body fluids in exercising humans. J. Appl. Physiol..

[15] Dodt C., Gunnarsson T., Elam M., Karlsson T., Wallin B.G. (1995). Central blood volume influences sympathetic sudomotor nerve traffic in warm humans. Acta Physiol. Scand..

[16] Mack G., Nishiyasu T., Shi X. (1995). Baroreceptor modulation of cutaneous vasodilator and sudomotor responses to thermal stress in humans. J. Physiol..

[17] Okazaki K., Ichinose T., Mitono H., Chen M., Masuki S., Endoh H., Hayase H., Doi T., Nose H. (2009). Impact of protein and carbohydrate supplementation on plasma volume expansion and thermoregulatory adaptation by aerobic training in older men. J. Appl. Physiol..

[18] Fortney S., Wenger C., Bove J., Nadel E. (1984). Effect of hyperosmolality on control of blood flow and sweating. J. Appl. Physiol..

[19] Barrera-Ramirez J., McGinn R., Carter R.M., Franco-Lopez H., Kenny G.P. (2014). Osmoreceptors do not exhibit a sex-dependent modulation of forearm skin blood flow and sweating. Physiol. Rep..

[20] Lynn A.G., Gagnon D., Binder K., Boushel R.C., Kenny G.P. (2012). Divergent roles of plasma osmolality and the baroreflex on sweating and skin blood flow. Am. J. Physiol. Regul. Integr. Comp. Physiol..

[21] Kamijo Y.-I., Ikegawa S., Okada Y., Masuki S., Okazaki K., Uchida K., Sakurai M., Nose H. (2012). Enhanced renal na+ reabsorption by carbohydrate in beverages during restitution from thermal and exercise-induced dehydration in men. Am. J. Physiol. Regul. Integr. Comp. Physiol..

[22] Tiwari S., Riazi S., Ecelbarger C.A. (2007). Insulin’s impact on renal sodium transport and blood pressure in health, obesity and diabetes. Am. J. Physiol. Renal Physiol..

[23] Suzuki A., Okazaki K., Imai D., Takeda R., Naghavi N., Yokoyama H., Miyagawa T. (2014). Thermoregulatory responses are attenuated after fructose but not glucose intake. Med. Sci. Sports Exerc..

[24] Cheuvront S.N., Bearden S.E., Kenefick R.W., Ely B.R., Degroot D.W., Sawka M.N., Montain S.J. (2009). A simple and valid method to determine thermoregulatory sweating threshold and sensitivity. J. Appl. Physiol..

[25] Hettinga F.J., Edwards A.M., Hanley B. (2019). The science behind competition and winning in athletics: Using world-level competition data to explore pacing and tactics. Front. Sports Act. Living.

[26] Jeukendrup A. (2014). A step towards personalized sports nutrition: Carbohydrate intake during exercise. Sports Med..

[27] Gagnon D., Kenny G.P. (2012). Does sex have an independent effect on thermoeffector responses during exercise in the heat?. J. Physiol..

[28] Inoue Y., Tanaka Y., Omori K., Kuwahara T., Ogura Y., Ueda H. (2005). Sex-and menstrual cycle-related differences in sweating and cutaneous blood flow in response to passive heat exposure. Eur. J. Appl. Physiol..

[29] Giersch G.E., Charkoudian N., Stearns R.L., Casa D.J. (2020). Fluid balance and hydration considerations for women: Review and future directions. Sports Med..

[30] Morris N.B., Chaseling G.K., Bain A.R., Jay O. (2019). Temperature of water ingested before exercise alters the onset of physiological heat loss responses. Am. J. Physiol. Regul. Integr. Comp. Physiol..

[31] Steinert R.E., Frey F., Töpfer A., Drewe J., Beglinger C. (2011). Effects of carbohydrate sugars and artificial sweeteners on appetite and the secretion of gastrointestinal satiety peptides. Br. J. Nutr..

[32] Sawka M.N., Burke L.M., Eichner E.R., Maughan R.J., Montain S.J., Stachenfeld N.S. (2007). American college of sports medicine position stand. Exercise and fluid replacement. Med. Sci. Sports Exerc..

[33] Dill D.B., Costill D.L. (1974). Calculation of percentage changes in volumes of blood, plasma, and red cells in dehydration. J. Appl. Physiol..

[34] Kraemer R.R., Brown B.S. (1986). Alterations in plasma-volume-corrected blood components of marathon runners and concomitant relationship to performance. Eur. J. Appl. Physiol. Occup. Physiol..

[35] Ramanathan N.L. (1964). A new weighting system for mean surface temperature of the human body. J. Appl. Physiol..

[36] Amano T., Fujii N., Kenny G.P., Nishiyasu T., Inoue Y., Kondo N. (2020). The relative contribution of α-and β-adrenergic sweating during heat exposure and the influence of sex and training status. Exp. Dermatol..

[37] Amano T., Fujii N., Inoue Y., Kondo N. (2018). Cutaneous adrenergic nerve blockade attenuates sweating during incremental exercise in habitually trained men. J. Appl. Physiol..

[38] Evans G.H., Shirreffs S.M., Maughan R.J. (2009). Postexercise rehydration in man: The effects of osmolality and carbohydrate content of ingested drinks. Nutrition.

[39] Borg G.A. (1982). Psychophysical bases of perceived exertion. Med. Sci. Sports Exerc..

[40] Gagge A.P., Stolwijk J.A., Hardy J.D. (1967). Comfort and thermal sensations and associated physiological responses at various ambient temperatures. Environ. Res..

[41] Gagnon D., Romero S.A., Ngo H., Poh P., Crandall C.G. (2016). Plasma hyperosmolality attenuates skin sympathetic nerve activity during passive heat stress in humans. J. Physiol..

[42] Jeukendrup A.E., Killer S.C. (2010). The myths surrounding pre-exercise carbohydrate feeding. Ann. Nutr. Metab..

[43] Thomas D., Brotherhood J., Brand J. (1991). Carbohydrate feeding before exercise: Effect of glycemic index. Int. J. Sports Med..

[44] Febbraio M.A., Keenan J., Angus D.J., Campbell S.E., Garnham A.P. (2000). Preexercise carbohydrate ingestion, glucose kinetics, and muscle glycogen use: Effect of the glycemic index. J. Appl. Physiol..

[45] Takamata A., Nose H., Kinoshita T., Hirose M., Itoh T., Morimoto T. (2000). Effect of acute hypoxia on vasopressin release and intravascular fluid during dynamic exercise in humans. Am. J. Physiol. Regul. Integr. Comp. Physiol..

[46] Chaseling G.K., Crandall C.G., Gagnon D. (2020). Skin blood flow measurements during heat stress: Technical and analytical considerations. Am. J. Physiol. Regul. Integr. Comp. Physiol..

